# Effect of Recycling and Autoclave Sterilization on the Unloading Forces of NiTi Closed-Coil Springs: An In Vitro Study

**Published:** 2013-12

**Authors:** Sh Momeni Danaei, M Oshagh, A Khozaei

**Affiliations:** a Dept. of Orthodontics, Member of Orthodontic Research Center, School of Dentistry, Shiraz University of Medical Sciences, Shiraz, Iran; b Orthodontist, Dept. of Orthodontics, International Branch, School of Dentistry, Shiraz University of Medical Sciences, Shiraz, Iran

**Keywords:** NiTi, Closed-coil Springs, Recycling

## Abstract

**Statement of Problem:** Clinicians use the NiTi coil springs frequently for its appropriate mechanical properties.

**Purpose:** The aim of this study was to determine the effect of recycling and autoclave sterilization on the unloading forces of NiTi closed coil springs.

**Materials and Method:** Fourteen NiTi closed coil spring with the length of 9mm were selected. Each coil was stretched to a peak extension of 12 mm. A universal testing machine was used to acquire load/deflection curve of the coil springs at 25±2°C. The influence of thermocycling (1000 cycles,5-55°C), autoclaving (134°C, 32PSI, 3min) and mechanical strain (9mm extension) which simulated the oral condition, were considered. Data were statistically analyzed by adopting Repeated Measures MANOVA Paired t-Test.

**Results: **Autoclaving in the 1, 4, 6 steps increased the force levels of coil springs about 2-5gf (*p*< 0.01). Thermocycling reduced their force levels about 4-6gf. Prolonged strain at 3, 5 steps decreased the magnitude of forces levels about 3-4gf.

**Conclusion: **Concerning all the limitations; according to the results of this study; it is possible to recycle Ni-Ti closed-coil springs without significant reduction in their force levels.

## Introduction

Ni-Ti alloys have been introduced to the orthodontics since early 1970s [1]. Their application has gradually become prevalent due to their appropriate mechanical properties, so that NiTi alloys have become more popular in the last decade [2]. Their main advantage are related to their nonlinear load deflection behavior; resulting their high flexibility, resiliency and spring back capability and low stiffness. They are relatively insensitive to the imprecise force adjustments in the clinic [3]. The application of NiTi coil springs has been popular in the fixed orthodontic treatment. They are fabricated in two forms: closed and open. They are used for space closure, protraction and retraction of teeth, applying force on the impacted teeth and so on [4]. They are more biocompatible than elastic chains because they are more consistent and they apply lower force levels on the teeth [5]. Space closure is performed more rapidly and constantly with the NiTi coil springs [6]. They provide physiologic and lower forces in the process of tooth movements when compared to the steel coil springs [7]. 

There are some disadvantages in the clinical application of NiTi alloys. One of these disadvantages is their high price. Occasionally they are 5 to 40 times more expensive than the other alloys [8]. Therefore, some clinicians have prompted to recycle NiTi wires [9]. About 52% of orthodontists in the United States recycle NiTi wires; although 55% of recyclers were concerned about the weakened mechanical properties of the recycled wires [10-11].

Some studies have evaluated the effects of thermocycling, mechanical loading and aging on NiTi coil springs and have found that the changes in the mechanical properties were clinically insignificant [2, 12] while another investigation showed that autoclaving did not affect the NiTi wires properties significantly [8]. A different clinical study demonstrated that sterilization slightly changed the load/deflection curves of NiTi wires but sterilization together with the clinical use reduced the mechanical properties significantly [9]. 

There are many studies about recycling of NiTi wires [8, 13-17]. While limited studies have been performed about the effects of sterilization and recycling on the mechanical properties of coil springs [2, 12, 18]. Considering the advantages of the coil springs and their high cost, their re-use after sterilization would be more prevalent and more economical. Hence, the purpose of this experimental study was to assess the effects of recycling and autoclave sterilization on the unloading forces of NiTi coil springs on the load/ deflection curve.

## Materials and Method

**Figure 1:**
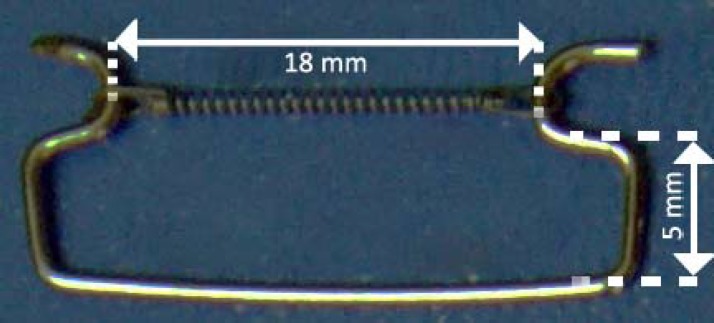
The coil spring is placed on the hooks

In this descriptive analytic experimental study, 14 NiTi closed coil springs (G & H; ccof9 XL, Greenwood, Canada) with the length of 9mm, wire diameter: 0.5mm, lumen diameter: 0.76mm, were selected. The samples were selected from 45 springs by adopting the simple randomized sampling method. A universal testing machine (ZwickRoell Z 020; Ulm, Germany) was used to acquire the load/deflection curve according to using the deflection rates of 5mm/min. The measurement was done at the room temperature of 25±2°C. The coil springs were mounted on the crossheads of the testing machine by using a pair of hooks made of 0.7 mm stainless steel wire (Figure 1). 

**Figure 2:**
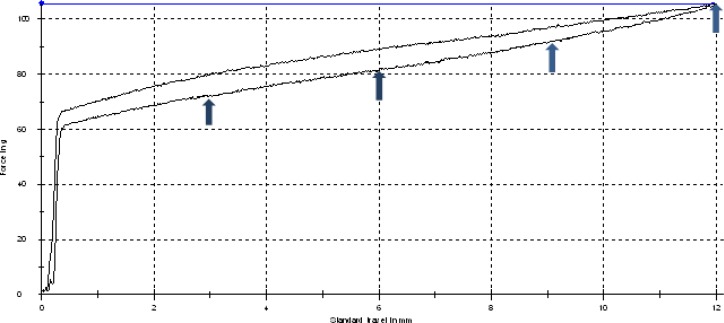
The forces was measured based on unloading part of load/deflection curve

At the beginning of the test, the hooks were separated equal to the coil spring’s length. The eyelets of coil springs were placed on the hooks and the measurement was started. Each coil stretched to a peak extension of 12mm. Then the deactivation stage was initiated and the machine crossheads approached each other. The load/ deflection curve was acquired during the tensile test. In this investigation, force levels were measured in 3, 6, 9, and 12 mm activation of coil springs based on the unloading part of the load deflection curve (Figure 2). The coils were investigated in the following steps:

**Control group:** After randomized selection of the samples, they were transferred to the machine and the forces were recorded. This information was considered as the control group.

**Step 1:** After the initial testing, the samples were placed in an open container and transferred to an autoclave (MOCOM; PRIMA, Australia). The coils were placed far from each to provide enough ventilation for a better sterilization. The process was done at 134°C and 32 psi for 3 minutes. After sterilization the samples were tested. The aim of this step was to evaluate the effects of autoclave sterilization on the NiTi coil spring’s unloading forces.

**Figure 3:**
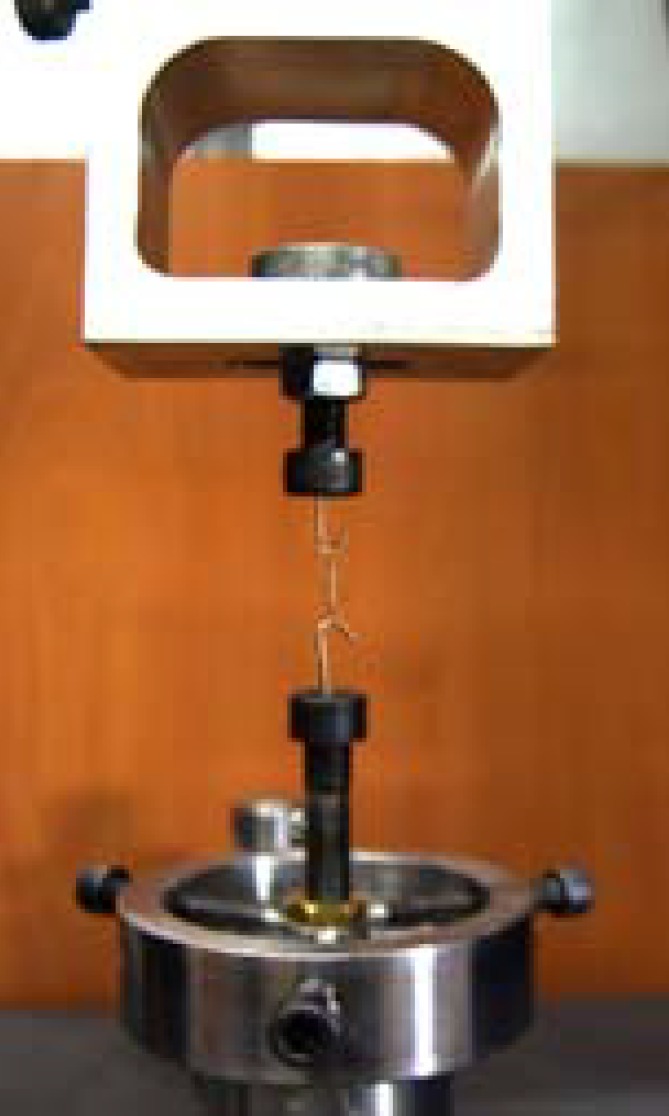
The coil spring is placed on the universal testing- machine’s cross heads

**Step** **2:** After step 1 the coil springs were stretched to 9mm extension and placed on two stainless- steel hooks (1mm in diameter) to maintain the coil’s extension (Figure 3). 

**Table 1:** The mean forces and standard deviations of coil springs in every step of the study

**Force levels** **12 mm in extension**		**Force levels** **9mm in extension**		**Force levels** **6 mm in extension**		**Force levels** **3 mm in extension**		**Control**
**Standard deviation**	**Mean**	**Standard** **deviation**	**Mean**	**Standard ** **deviation**	**Mean**	**Standard ** **deviation**	**Mean**	
2.17	107.09	2.49	92.59	2.17	83.10	2.26	75.08	
1.86	110.85	1.89	97.29	1.67	88.46	1.57	79.81	1
1.66	106.04	1.74	92.63	1.95	82.97	1.88	74.64	2
2.47	102.16	2.18	88.78	1.87	79.12	1.77	70.93	3
2.17	104.46	2.26	90.59	1.71	81.60	1.93	73.89	4
2.68	100.57	1.96	87.14	1.82	77.76	2.28	68.86	5
2.28	103.05	1.90	89.60	1.90	80.27	1.92	78.8	6

A caliper with the measurement accuracy of 0.01 mm (Hygeia Dental Co. Ltd; Hong Kong) was used to measure the distance between the two hooks and the amount of coil springs activation. After placement of the coil springs on the hooks, the distance between the hooks were measured again and any corrections in the distance were implemented. Then the samples were subjected to thermocycling in 1000 cycles from 5°C to 55°C. The springs were immersed in cooling and heating bath for 1 minute consecutively with 2±1 seconds interval time between the immersions in which they were kept out of the bath at air temperature. At the end of thermocycling, the samples were tested immediately by the machine and the data were recorded. The purpose of this step was to evaluate the effects of temperature variations; in which occurs in the mouth, on NiTi coil springs unloading forces.

**Step 3:** As with step 2, the samples were placed to the same hooks again and activated for 9 mm. Then they immersed in a container of Normal Saline (Sodium Chloride 0.9 %) and the container was placed in an incubator (NUVE; EN 250, Turkey) at 37°C for 1 month. Then, the samples were removed and tested.

The aim of this step was to evaluate the effects of 1 month strain, which is comparable to one cycle of application in a patient’s mouth, on NiTi coil springs unloading forces.

**Step 4:** After testing in step 3, the samples were sterilized by the autoclave (the same as step 1) and were tested. The purpose of this step was to evaluate the effects of two periods of autoclave sterilization on NiTi coil springs unloading forces.

**Step 5:** As in step 2, the samples were placed on the hooks again and incubated at 37°C and after 1 month they were tested. The aim of this step was to evaluate the effects of two-month strain, which is comparable to two cycles of application in patient’s mouth, on NiTi coil springs unloading forces.

**Step 6:** As in step 1, the samples were sterilized by the autoclave then were tested. The purpose of this step was to evaluate the effects of three cycles of auto-clave sterilization on NiTi coil springs unloading forces.

In the all steps described above, measurements were done by one technician who was blind to the steps of procedures performed on the samples.

Data were analyzed by statistical tests of Repeated Measures MANOVA Paired t- Test.

## Results

Table 1 displays the mean forces and standard deviations of coil springs in every steps of the study.

Table 2 presents the p-values of different group comparisons. Results showed that in the first step of treatment, the loads exerted by the coil springs were significantly increased (*p*< 0.01) and the mean increase was measured to be in the range of 3-5 gram force (gf) (3-5%). In the second stage, thermocycling decreased the loads (4-7%) to the level of the control group with no significant difference in comparison with control group. After being loaded for 1 month during the third stage, the force level decreased (3-5%) to lower than the control group. Autoclave sterilization in the fourth stage increased the loads (2-4%) to the level of the control group. The second one-month cycle during the fifth stage, resulted in significant reduction (3-6%) in the force level of the coil springs. Autoclaving in the sixth stage increased the magnitude of forces (2-4%) but to a lower level, compared with control group.

**Table 2:** The p-values of different group comparisons

**P. value** **12mm in extension**	**P. value** **9 mm in extension**	**P. value** **6 mm in extension**	**P. value** **3mm in extension**	**Control**
0.000 0.205 0.000 0.010 0.000 0.000	0.000 0.963 0.003 0.047 0.000 0.003	0.000 0.876 0.001 0.049 0.000 0.003	0.000 0.623 0.000 0.150 0.000 0.002	1 2 3 4 5 6
0.000 0.000 0.000 0.000 0.000 0.000	0.000 0.000 0.000 0.000 0.000 0.000	0.000 0.000 0.000 0.000 0.000 0.000	0.000 0.000 0.000 0.000 0.000 0.000	1 2 3 4 5 6
0.205 0.000 0.000 0.077 0.000 0.001	0.963 0.000 0.000 0.049 0.000 0.000	0.876 0.000 0.000 0.098 0.000 0.000	0.623 0.000 0.000 0.376 0.000 0.000	2 1 3 4 5 6
0.000 0.000 0.000 0.029 0.001 0.000	0.003 0.000 0.000 0.085 0.022 0.178	0.003 0.000 0.000 0.005 0.033 0.087	0.000 0.000 0.000 0.002 0.005 0.113	3 1 2 4 5 6
0.10 0.000 0.077 0.032 0.001 0.110	0.047 0.000 0.049 0.085 0.002 0.260	0.049 0.000 0.098 0.005 0.000 0.075	0.150 0.000 0.376 0.002 0.000 0.023	4 1 2 3 5 6
0.000 0.000 0.000 0.029 0.001 0.000	0.000 0.000 0.000 0.022 0.002 0.000	0.000 0.000 0.000 0.033 0.000 0.000	0.000 0.000 0.000 0.005 0.000 0.000	5 1 2 3 4 6
0.000 0.000 0.001 0.202 0.110 0.000	0.003 0.000 0.000 0.178 0.260 0.000	0.003 0.000 0.000 0.087 0.075 0.000	0.002 0.000 0.000 0.113 0.523 0.000	6 1 2 3 4 5

## Discussion

14 Ni-Ti closed-coil springs were analyzed in this *in-vitro *study. The spring’s force levels were measured at 25° C. In several studies, the forces were measured at 37°C using thermal bath in order to simulate oral conditions as close as possible [2, 18-19]. In the present study, the thermal bath was not available to mount on the testing machine. The objective of this study was to assess the changes in the spring forces thus the study was designed based on the same condition in the control and the treatment groups at 25°C. 

In order to measure the springs’ force levels, the unloading part of the load/deflection curve was used. Since in the clinical situations, the springs exert their forces in the unloading phase; the unloading forces of coil springs help us understand what might happen in the real clinical situation [3]. 

According to the present study, autoclave sterilization in all three steps (steps 1, 4, 6) resulted in an increase in the springs’ force levels. This is in agreement with the findings of the study of Ramazanzadeh et al. [20] which was performed on super elastic Ni-Ti wires. In this study the wires were subjected to strain for two months that resulted in a decrease in their forces, but after autoclaving, their forces increased [20, 21]. However in two other studies [3, 8] autoclaving resulted in a reduction in the Ni-Ti wire force levels. The reason for this difference might be due to the fact that Ni-Ti coil springs are extremely sensitive to changes in the environmental temperature; the amount of force generated by the springs depends on the transformation temperature [22]. Barwart reported that the force level of Ni-Ti closed-coil spring at 37°C is twice more than 20°C [23]. 

At lower temperature; NiTi alloys are completely in the martensitic phase [7]. The increase in temperature causes their gradual transformation into austenitic phase that consequently leads to increase in forces magnitude [7]. Through the process of autoclaving, the coil springs tend to transform to the austenitic phase and gradually, with the decrease in the temperature, the phase should change into the martensite phase. Some phase transformations, from austenite to martensite in the Ni-Ti alloys, can occur by stress application which is called stress- induced martensite (SIM). According to Wichelhause et al. investigation [2] a considerable deflection is necessary to form enough SIM therefore they recommended performing some pre-activation before testing. Since the springs were tested without pre-activation in our study, it is possible that SIM could not have happened and austenitic phase would have been predominant in the springs at the time of the test. An increase in the spring forces after autoclaving is the result since the forces were recorded exactly at the time of deflection and probably SIM have not been occurred. In the current study if the springs were subjected to some pre-activation before the test, the results would have been possibly more precise. It is recommended to consider this issue in the future studies.

In this study, thermocycling reduced the forces of the springs. Wichelhause et al. [2] showed that thermocycling resulted in a 15-20% increase in the force levels. The reason for this difference might be related to the differences in the study designs and methods. In the present study, the springs were subjected to thermocycling after the process of autoclaving, while in the study of Wichelhause et al. [2], the autoclave was not employed. Sterilization in the temperatures higher than 60° C can create changes in the crystalline structure of Ni-Ti alloy and subsequently lead to changes in the mechanical properties of them [24]. Lijima et al. [25] also evaluated the influence of temperature on the mechanical properties of Ni-Ti wires. They demonstrated that the increase in the temperature raises the force levels and the decrease in the temperature reduces it, however, the force levels after thermocycling was increased. They concluded that sequential heating-cooling cycles may result in higher the force levels. Vidoni et al. [12] showed that prolonged strain and thermocycling does not noticeably change their force levels. In their study, the effect of thermo cycling was not evaluated separately and their findings showed the effect of thermo cycling and loading simultaneously.

In the fourth and the sixth stages of the present study; after being exposed to strain for one month, force levels of the springs decreased. Although this decrease was statistically significant, the mean reduction in forces at different tensions was in the range of 4-7 gf which is clinically insignificant. In other studies, subjecting the Ni-Ti closed-coil springs to prolonged strain resulted in a decrease in their force magnitude which was not clinically significant [2, 5, 15, 26]. Biermann et al. [26] evaluated phase transformation of copper Ni-Ti wires before and after their clinical usage and showed similar phase transformation before and after their application.

According to the findings of this research; it is possible to recycle Ni-Ti closed-coil springs without devising significant reduction in their force levels. Future studies are solicited to support the findings of the current study.

**Limitations of the study**

In this study the springs were immersed in Sodium Chloride solution for incubation. The application of artificial saliva would more precisely simulate the clinical situation. 

In this study, due to the unavailability of a thermal bath that could be mountable on the testing machine, the test could not be performed at an environment containing 37°C water. If the test could be performed under such a condition, the results would possibly be more relevant to the clinical situations.

Given that few studies are performed on the recycling of Ni-Ti closed-coil springs, more research in this field are recommended, particularly evaluating the effect of autoclaving on phase transformations of these coil springs.

## Conclusion

Regarding the limitations of the current study we can conclude from this study that:

1. Autoclave sterilization can increase the force levels of Ni-Ti closed-coil springs.

2. Thermocycling can decrease the force levels of Ni-Ti closed-coil springs.

3. The recycling of the Ni-Ti closed coil springs can decrease their force levels but this is not clinically significant.

According to the findings of this study; it is possible to recycle Ni-Ti closed-coil springs without significant reduction in their force levels but more adherent studies in this subject are recommended in the future.

## References

[1] Han S, Quick DC (1993). Nickel-titanium spring properties in a simulated oral environment. Angle Orthod.

[2] Wichelhaus A, Brauchli L, Ball J, Mertmann M (2010). Mechanical behavior and clinical application of nickel-titanium closed-coil springs under different stress levels and mechanical loading cycles. Am J Orthod Dentofacial Orthop.

[3] Kapila S, Reichhold GW, Anderson RS, Watanabe LG (1991). Effects of clinical recycling on mechanical properties of nickel-titanium alloy wires. Am J Orthod Dentofacial Orthop.

[4] Samuels RH, Peak JD (1998). Use of nickel titanium closed-coil springs to align unerupted teeth: a case report. Am J Orthod Dentofacial Orthop.

[5] Bennett JC, McLaughlin RP (1990). Controlled space closure with a preadjusted appliance system. J Clin Orthod.

[6] Samuels RH, Rudge SJ, Mair LH (1993). A comparison of the rate of space closure using a nickel-titanium spring and an elastic module: a clinical study. Am J Orthod Dentofacial Orthop.

[7] Santoro M, Nicolay OF, Cangialosi TJ (2001). Pseudoelasticity and thermoelasticity of nickel-titanium alloys: a clinically oriented review Part II: Deactivation forces. Am J Orthod Dentofacial Orthop.

[8] Alavi S, Raji SH, Ghorbani AA (2009). Effects of steam and dry-heat sterilization on bending properties of NiTi wires. Orthodontic Waves.

[9] Kapila S, Haugen JW, Watanabe LG (1992). Load-deflection characteristics of nickel-titanium alloy wires after clinical recycling and dry heat sterilization. Am J Orthod Dentofacial Orthop.

[10] Buckthal JE, Mayhew MJ, Kusy RP, Crawford JJ (1986). Survey of sterilization and disinfection procedures. J Clin Orthod.

[11] Cash RG (1990). Trends in sterilization and disinfection procedures in orthodontic offices. Am J Orthod Dentofacial Orthop.

[12] Vidoni G, Perinetti G, Antoniolli F, Castaldo A, Contardo L (2010). Combined aging effects of strain and thermocycling on unload deflection modes of nickel-titanium closed-coil springs: an in-vitro comparative study. Am J Orthod Dentofacial Orthop.

[13] Oshagh M, Hematiyan MR, Mohandes Y, Oshagh MR, Pishbin L (2012). Autoclaving and clinical recycling: effects on mechanical properties of orthodontic wires. Indian J Dent Res.

[14] Alcock JP, Barbour ME, Sandy JR, Ireland AJ (2009). Nanoindentation of orthodontic archwires: The effect of decontamination and clinical use on hardness, elastic modulus and surface roughness. Dent Mater.

[15] Lee SH, Chang YI (2001). Effects of recycling on the mechanical properties and the surface topography of nickel-titanium alloy wires. Am J Orthod Dentofacial Orthop.

[16] Oshagh M, Ajami S (2010). A comparison of force decay: elastic chain or tie-back method?. World J Orthod.

[17] Maganzini AL, Wong AM, Ahmed MK (2010). Forces of various nickel titanium closed coil springs. Angle Orthod.

[18] Smith GA, von Fraunhofer JA, Casey GR (1992). The effect of clinical use and sterilization on selected orthodontic arch wires. Am J Orthod Dentofacial Orthop.

[19] Staggers JA, Margeson D (1993). The effects of sterilization on the tensile strength of orthodontic wires. Angle Orthod.

[20] Ramazanzadeh BA, Ahrari F, Sabzevari B, Zebarjad SM, Ahrari A (2011). Effects of a simulated oral environment and sterilization on load-deflection properties of superelastic nickel titanium-based orthodontic wires. Int J Orthod Milwaukee.

[21] Espinar-Escalona E, Llamas-Carreras JM, Barrera-mora JM, Abalos-lasbrucci CA, Gil-Mur FJ (2013). Effect of temperature on the orthodontic clinical application of niti closed-coil springs. Med Oral Patol Oral Cir Bucal.

[22] Pandis N, Bourauel CP, Tomobe S (2010). Nickel-titanium (Ni Ti) arch wires: the clinical significance of super elasticity. Semin Orthod.

[23] Barwart O (1996). The effect of temperature change on the load value of Japanese NiTi coil springs in the superelastic range. Am J Orthod Dentofacial Orthop.

[24] Miura F, Mogi M, Ohura Y, Hamanaka H (1986). The super-elastic property of the Japanese NiTi alloy wire for use in orthodontics. Am J Orthod Dentofacial Orthop.

[25] Biermann MC, Berzins DW, Bradley TG (2007). Thermal analysis of as-received and clinically retrieved copper-nickel-titanium orthodontic archwires. Angle Orthod.

[26] Iijima M, Ohno H, Kawashima I, Endo K, Mizoguchi I (2002). Mechanical behavior at different temperatures and stresses for superelastic nickel-titanium orthodontic wires having different transformation temperatures. Dent Mater.

[27] Nattrass C, Ireland AJ, Sherriff M (1998). The effect of environmental factors on elastomeric chain and nickel titanium coil springs. Eur J Orthod.

[28] Angolkar PV, Arnold JV, Nanda RS, Duncanson MG Jr (1992). Force degradation of closed coil springs: an in vitro evaluation. Am J Orthod Dentofacial Orthop.

